# Supplementary figures and images for: Alternative lipid synthesis in response to phosphate limitation promotes antibiotic tolerance in Gram-negative ESKAPE pathogens
Source: PLoS Pathog. 2025 Feb 7;21(2):e1012933. doi: 10.1371/journal.ppat.1012933 (PMC11828411; doi:10.1371/journal.ppat.1012933)

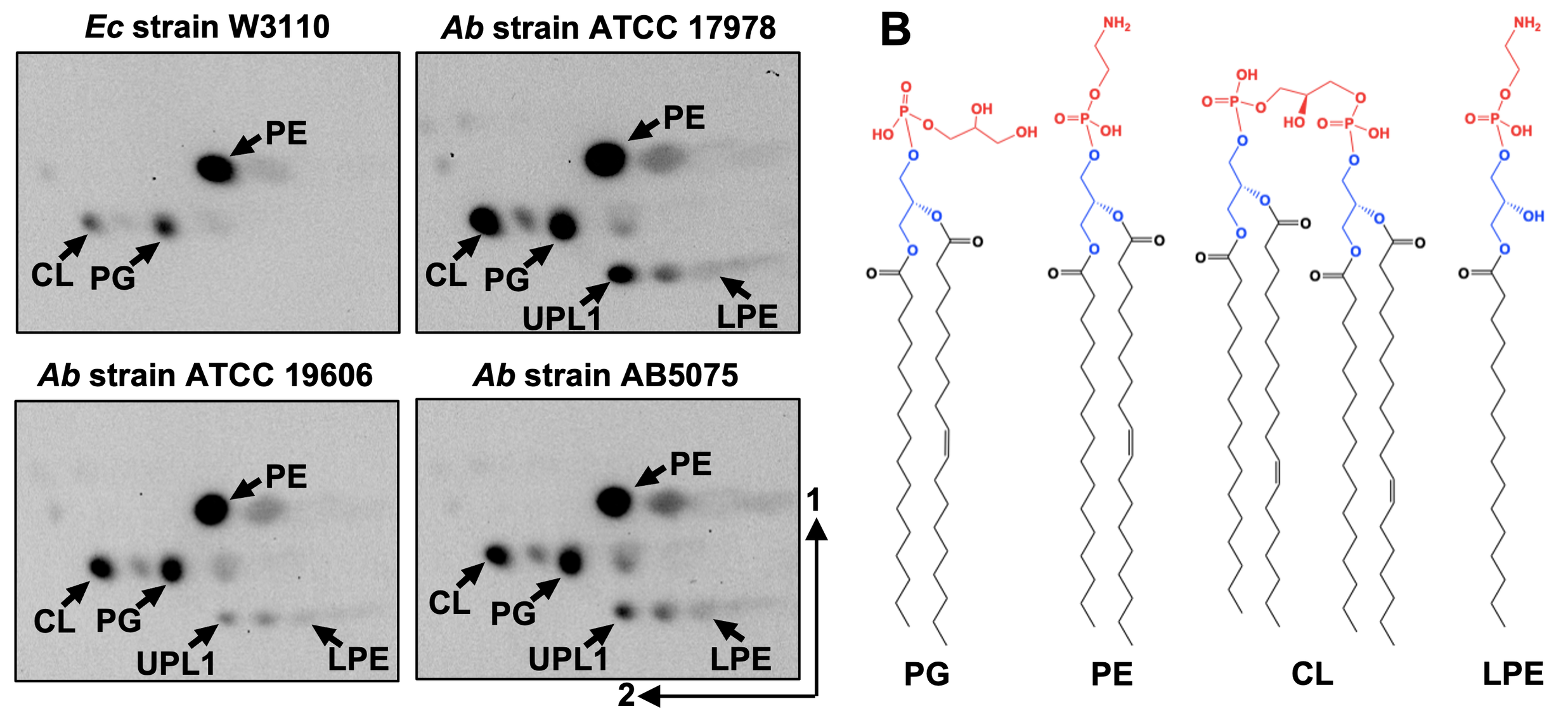

Supplement: S1 Fig — A. Strains were grown in complex media (LB broth) in the presence of 32P-orthopohosphoric acid until mid-logarithmic growth phase. Cells were collected and total lipids were extracted using the Bligh and Dyer method and separated using 2-dimensional thin-layer chromatography. Labelled lipids include anionic cardiolipin (CL) and phosphatidylglycerol (PG), aminolipids phosphatidylethanolamine (PE) and lyso-PE (LPE), and unknown phospholipid 1 (UPL1). B. PG, PE, CL and LPE chemical structures. Head groups are red, the glycerol backbone is blue, and fatty acids are black. (TIF) [file ppat.1012933.s001.tif]

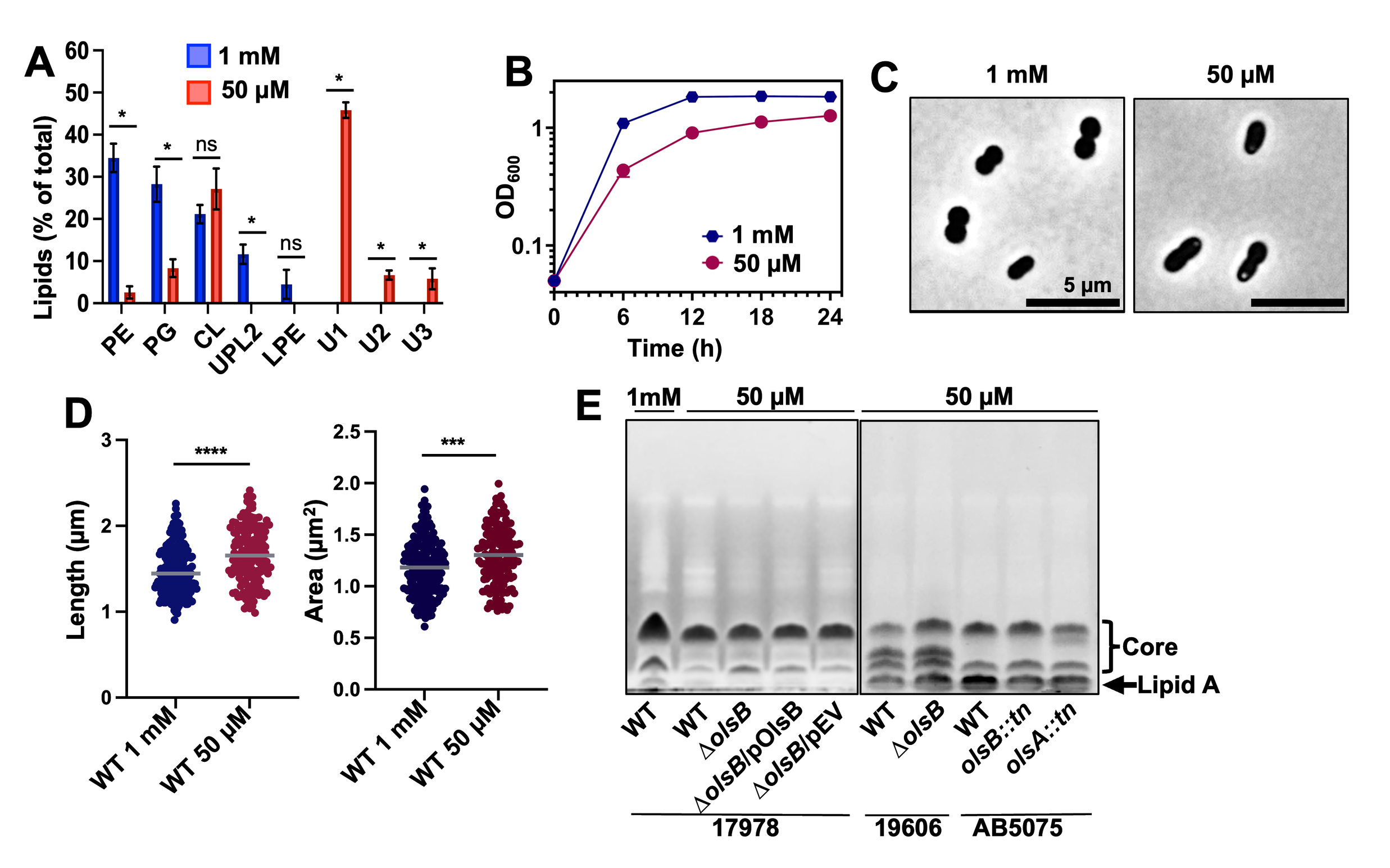

Supplement: S2 Fig — A. Quantification of total lipid in TLC Fig 1 grown in excess or limiting phosphate concentrations. Lipids are graphed as a percentage of the total. Significance testing conducted using Student t test with two-tailed distribution assuming equal variance. Lines indicate standard deviation. *P < 0.05; ns is not significant. B. A. baumannii ATCC 17978 was cultured in minimal medium containing either excess (1 mM) or limiting (50 µM) phosphate for 24 hours. C. Phase-contrast images of A. baumannii grown under these conditions, captured during the exponential phase. Scalebar is 10 µm. D. Cell length and area were quantified for each population (n ≥ 150) using ImageJ software. Each point represents an individual cell. The experiment was repeated twice, and one representative dataset was reported. Significance testing conducted using Student t test with two-tailed distribution assuming equal variance. ***P < 0.001, ****P < 0.0001. E. Proteinase K-treated whole-cell lysate from wild-type A. baumannii strains ATCC 17978, ATCC 19606, and AB5075, as well as from aminolipid-deficient mutants grown under excess (1mM) or limiting (50 µM) phosphate conditions. The LOS samples were separated using SDS-PAGE and stained using Pro-Q Emerald 300. (TIF) [file ppat.1012933.s002.tif]

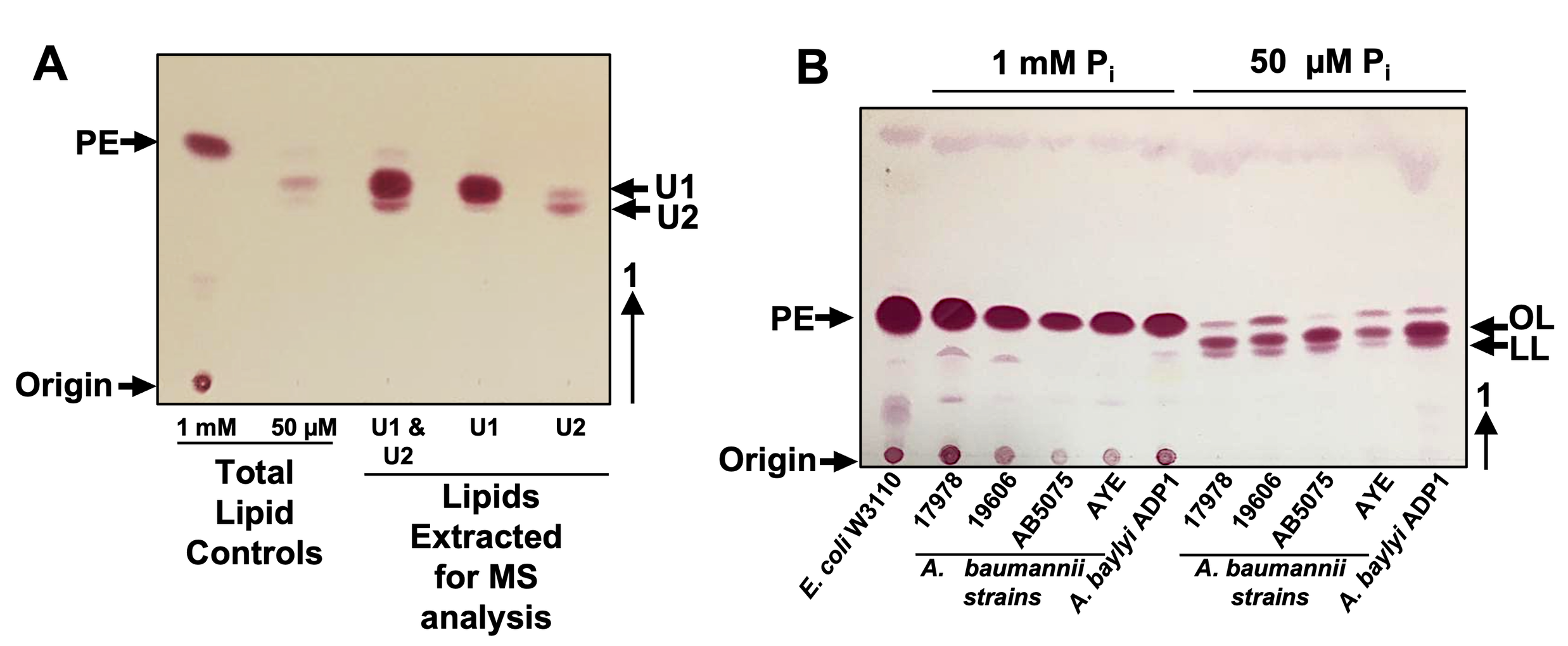

Supplement: S3 Fig — A. To produce A. baumannii lipid samples for MS analysis, total lipids were separated using thin-layer chromatography and scraped from the plate, extracted using the Bligh and Dyer method, and run alongside lipid controls. Extractions resulted in isolation of U1 & U2, U1, or U2. Lipids were stained with ninhydrin to visualize aminolipids. Specific lipids are labelled: PE, phosphatidylethanolamine; U1, unknown lipid 1; U2, unknown lipid 2. B. Cells were grown in minimal medium supplemented with excess (1mM) or limiting (50 µM) phosphate conditions from indicated Acinetobacter strains. Total lipids were extracted from cells grown in media with limiting phosphate concentrations. Total lipids were spotted on thin-layer chromatography and separated based on hydrophobicity. Plates were stained with ninhydrin to visualize aminolipids. Specific lipids are labelled: PE, phosphatidylethanolamine; OL, ornithine lipid; LL, lysine lipid. (TIF) [file ppat.1012933.s003.tif]

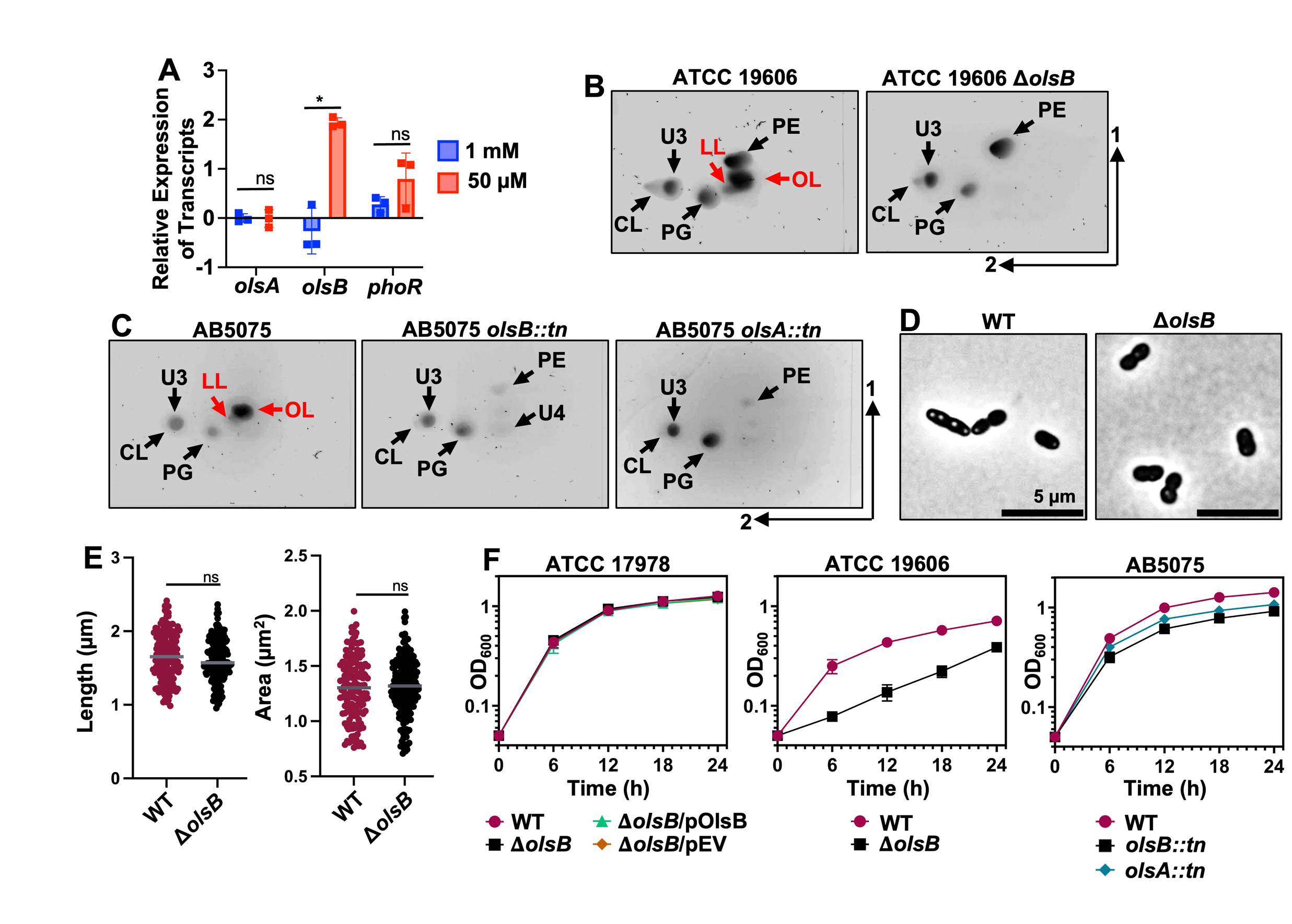

Supplement: S4 Fig — A. Relative-abundance quantitative PCR (qPCR) of genes after A. baumannii growth in excess or limiting phosphate concentrations (n = 3). Lines indicate standard deviation. Significance testing was conducted using Student t test with two-tailed distribution assuming equal variance. *P < 0.05; ns = not significant. B. 2D thin-layer chromatography lipid analysis in ATCC 19606 wild type and the ΔolsB mutant strain after growth in limiting (50 µM) phosphate concentrations. Lipids were stained with sulfuric acid. C. Analysis in AB5075 wild type and transposon (Tn101) mutant strains after growth in limiting (50 µM) phosphate concentrations. Specific lipids are labelled: PE, phosphatidylethanolamine; PG, phosphatidylglycerol; CL, cardiolipin; OL, ornithine lipid; LL, lysine lipid. OL and LL aminolipids are labelled in red. D. Phase-contrast images of A. baumannii strain ATCC 17978 grown under phosphate limiting conditions, captured during exponential phase growth. Scalebar is 10 µm. E. Cell length and area of strain ATCC 17978 were quantified for each population (n ≥ 150) using ImageJ software. Each point represents an individual cell. Significance testing was conducted using Student t test with two-tailed distribution assuming equal variance. ns = not significant. F. Optical density (OD600) measurements of A. baumannii strains grown in 50 µM phosphate over 24 h. (TIF) [file ppat.1012933.s004.tif]

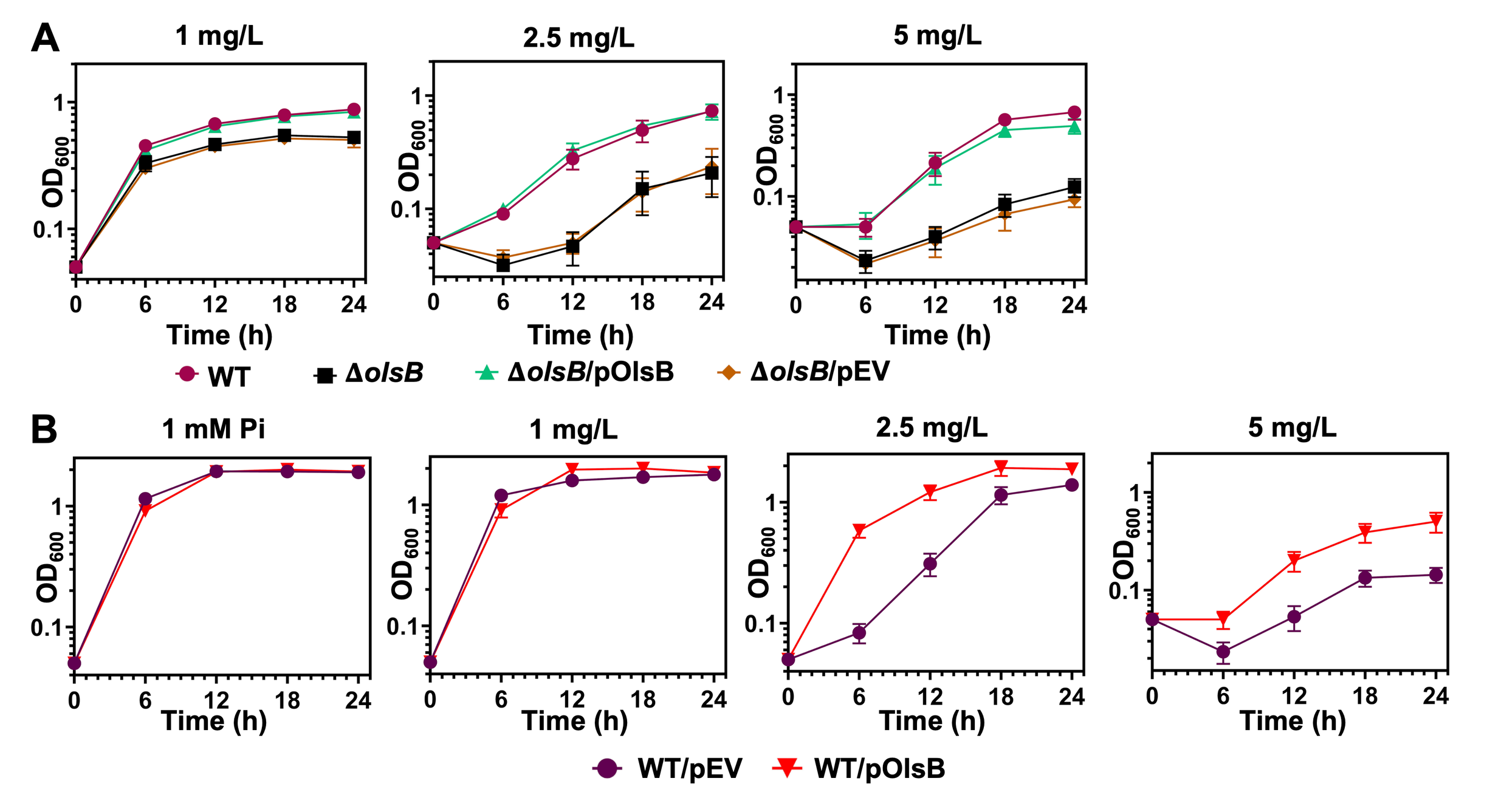

Supplement: S5 Fig — Growth (OD600) of A. baumannii ATCC 17978 strains was measured at 37 °C in minimal medium with limiting phosphate (A) or excess phosphate (B), and in the presence of colistin at concentrations of 1, 2.5, or 5 mg/L. (TIF) [file ppat.1012933.s005.tif]

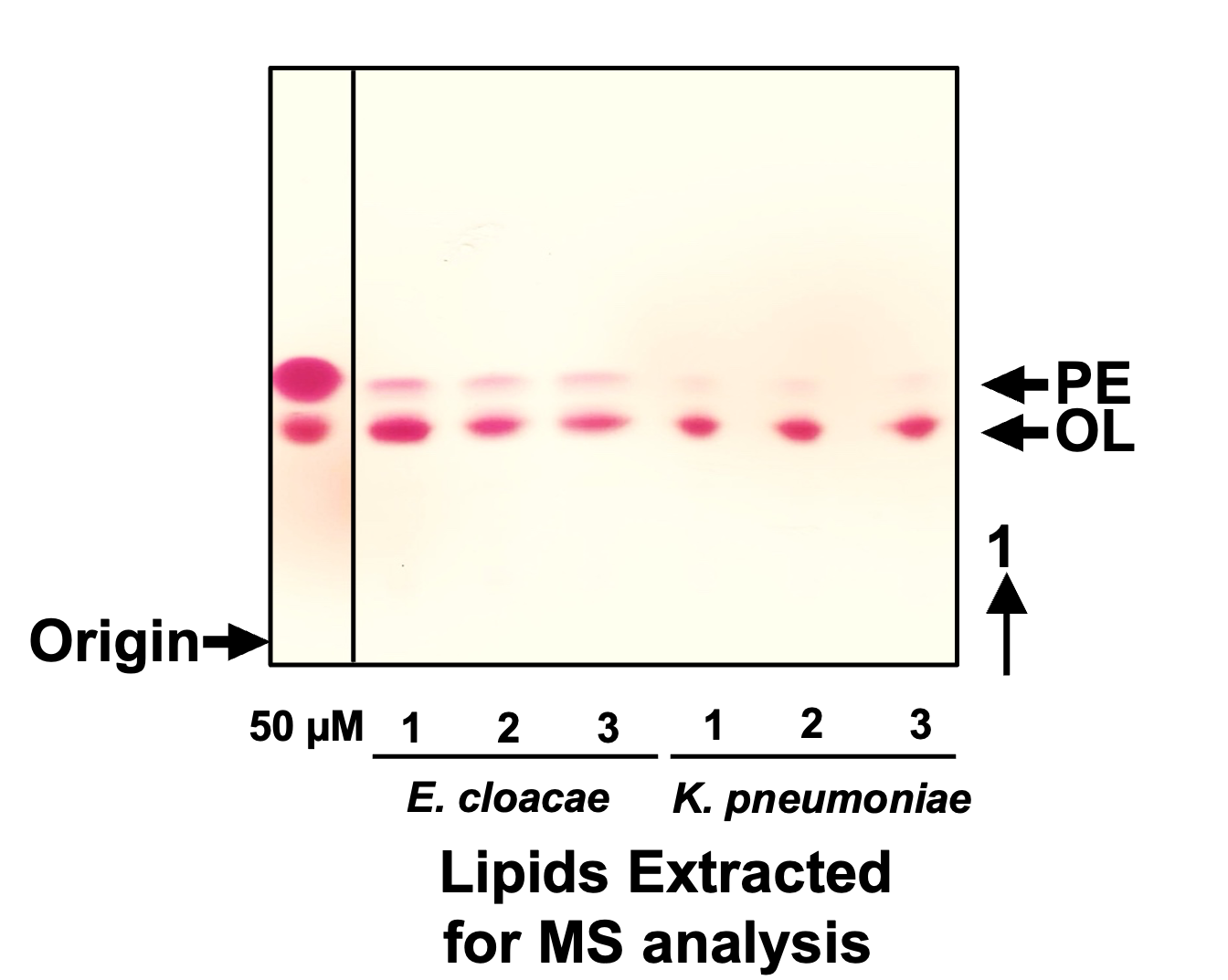

Supplement: S6 Fig — To produce E. cloacae and K. pneumoniae samples for MS analysis, total lipids were extracted from cells grown in media with limiting phosphate concentrations. Total lipids were separated using thin-layer chromatography and scraped from the plate, extracted using the Bligh and Dyer method, and run alongside lipid controls. Lipids were stained with ninhydrin to visualize aminolipids. Specific lipids are labelled: PE, phosphatidylethanolamine; OL; ornithine lipid (predicted). (TIF) [file ppat.1012933.s006.tif]
