# Supplementary material for: Alternative lipid synthesis in response to phosphate limitation promotes antibiotic tolerance in Gram-negative ESKAPE pathogens
Source: PLoS Pathog. 2025 Feb 7;21(2):e1012933. doi: 10.1371/journal.ppat.1012933 (PMC11828411; doi:10.1371/journal.ppat.1012933)
Supplement: S6 Table — (DOCX) [file ppat.1012933.s012.docx]

**Table S6: Strains and plasmids used in this study.**

| **Strain or Plasmid** | **Description** | **Reference or Source** |
| --- | --- | --- |
| **Strains** |  |  |
| *A. baumannii* ATCC 17978 | Wild type | (1) |
| *A. baumannii* ATCC 19606 | Wild type | (2) |
| *A. baumannii* AB5075 | Wild type | (3) |
| *A. baumannii* AYE | Wild type | (4) |
| *A. baylyi* ADP1 | Wild type | (5) |
| *P. aeruginosa* PAO1 | Wild type | (6) |
| *K. pneumoniae* KPNIH1 | Wild type | (7) |
| *E. cloacae* ATCC 13047 | Wild type | (8) |
| *A. baumannii* ATCC 17978 | *ΔolsB (A1S_0889)::deletion* | This study |
| *A. baumannii* ATCC 17978 | *ΔphoR (A1S_3376)::deletion* | This study |
| *A. baumannii* ATCC 19606 | *ΔolsB (HMPREF0010_01383)::deletion* | This study |
| *A. baumannii* AB5075 | *ΔolsB (ABUW_3039)::tn101* | (9) |
| *A. baumannii* AB5075 | *ΔolsA (ABUW_0502)::tn101* | (9) |
| *A. baumannii* AB5075 | *ΔphoR (ABUW_0105)::tn26* | (9) |
| *E. coli* W3110 | Wild type*,* F*- λ-, rph-1 IN (rrnD, rrnE)1* | (10) |
| *E. coli* DH5α | recA1, ϕ80 *lacZ*ΔM15, host for cloning | (11) |
| **Plasmids** |  |  |
| pAT03 | pMMB67EH with FLP recombinase, Tet^R^ | (12) |
| pAT04 | pMMB67EH with REC*_Ab_* system, Tet^R^ | (12) |
| pKD4 | Kan^R^ | (13) |
| pMMB67EHKn | pMMB67EH with the Kan^R^ gene from pKD4 inserted into the PvuI site, Kan^R^ | (14) |
| pROB01 | pMMB67EHKn carrying *olsB* (*A1S_0889*) | This study |
| pROB02 | pMMB67EHKn carrying *phoR* (*A1S_3376*) | This study |

**References**

1. Baumann P, Doudoroff M, Stanier RY. A study of the Moraxella group. II. Oxidative-negative species (genus *Acinetobacter*). *J Bacteriol*. 1968 May;95(5):1520–41.

2. Bouvet PJM, Grimont PAD. Taxonomy of the Genus Acinetobacter with the Recognition of *Acinetobacter baumannii sp. nov., Acinetobacter haemolyticus sp. nov., Acinetobacter johnsonii sp. nov., and Acinetobacter junii sp. nov.* and Emended Descriptions of *Acinetobacter calcoaceticus* and *Acinetobacter lwoffii*. I*nternational Journal of Systematic and Evolutionary Microbiology*. 1986;36(2):228–40.

3. Jacobs AC, Thompson MG, Black CC, Kessler JL, Clark LP, McQueary CN, et al. AB5075, a Highly Virulent Isolate of *Acinetobacter baumannii*, as a Model Strain for the Evaluation of Pathogenesis and Antimicrobial Treatments. *mBio*. 2014 Jul 1;5(3):e01076-14.

4. Fournier PE, Vallenet D, Barbe V, Audic S, Ogata H, Poirel L, et al. Comparative genomics of multidrug resistance in *Acinetobacter baumannii. PLoS Genet*. 2006 Jan;2(1):e7.

5. Barbe V, Vallenet D, Fonknechten N, Kreimeyer A, Oztas S, Labarre L, et al. Unique features revealed by the genome sequence of *Acinetobacter sp.* ADP1, a versatile and naturally transformation competent bacterium. *Nucleic Acids Res*. 2004;32(19):5766–79.

6. Stover CK, Pham XQ, Erwin AL, Mizoguchi SD, Warrener P, Hickey MJ, et al. Complete genome sequence of *Pseudomonas aeruginos*a PAO1, an opportunistic pathogen. *Nature*. 2000 Aug 31;406(6799):959–64.

7. Snitkin ES, Zelazny AM, Thomas PJ, Stock F, NISC Comparative Sequencing Program Group, Henderson DK, et al. Tracking a hospital outbreak of carbapenem-resistant *Klebsiella pneumoniae* with whole-genome sequencing. *Sci Transl Med*. 2012 Aug 22;4(148):148ra116.

8. Ren Y, Ren Y, Zhou Z, Guo X, Li Y, Feng L, et al. Complete genome sequence of *Enterobacter cloacae* subsp. cloacae type strain ATCC 13047. *J Bacteriol*. 2010 May;192(9):2463–4.

9. Gallagher LA, Ramage E, Weiss EJ, Radey M, Hayden HS, Held KG, et al. Resources for Genetic and Genomic Analysis of Emerging Pathogen *Acinetobacter baumannii. J Bacteriol*. 2015 Jun;197(12):2027–35.

10. Bachmann BJ. Pedigrees of some mutant strains of *Escherichia coli* K-12. *Bacteriol Rev*. 1972 Dec;36(4):525–57.

11. Hanahan D. Studies on transformation of *Escherichia coli* with plasmids. *J Mol Biol*. 1983 Jun 5;166(4):557–80.

12. Tucker AT, Nowicki EM, Boll JM, Knauf GA, Burdis NC, Trent MS, et al. Defining Gene-Phenotype Relationships in *Acinetobacter baumannii* through One-Step Chromosomal Gene Inactivation. *mBio*. 2014 Aug 29;5(4).

13. Datsenko KA, Wanner BL. One-step inactivation of chromosomal genes in *Escherichia coli* K-12 using PCR products. *Proceedings of the National Academy of Sciences*. 2000 Jun 6;97(12):6640–5.

14. Kang KN, Kazi MI, Biboy J, Gray J, Bovermann H, Ausman J, et al. Septal Class A Penicillin-Binding Protein Activity and ld-Transpeptidases Mediate Selection of Colistin-Resistant Lipooligosaccharide-Deficient *Acinetobacter baumannii. mBio*. 2021 Jan 5;12(1):e02185-20.
