# Supplementary material for: Alternative lipid synthesis in response to phosphate limitation promotes antibiotic tolerance in Gram-negative ESKAPE pathogens
Source: PLoS Pathog. 2025 Feb 7;21(2):e1012933. doi: 10.1371/journal.ppat.1012933 (PMC11828411; doi:10.1371/journal.ppat.1012933)
Supplement: S7 Table — (DOCX) [file ppat.1012933.s013.docx]

**Table S7: Primers used in this study.**

| **Oligo Name** | **Sequence (5’ to 3’)** |
| --- | --- |
| **Deletion Primers** |  |
| 17978 *olsB*Ab Kan FRT 5' | AGTGGGTGTCACTAGGAGCGTTCATTATGCTGGAAAAATTTAATCAATATCGCCAAACCTGGACTTTACCTTTAAATCGCCATAAGGCTAACAATCAAACACAATTCCGCTTTGAATGGGTTGATAGCGATTGTGTAGGCTGGAGCTGCTTCG |
| 17978 *olsB*Ab Kan FRT 3' | TTGTTCATTACAAACGAAGTGGCAATTTTATTCACTTCTAAAAATACAAAGTAATCGAGACAGTTAAATTCAGCATCAAAGAAAGCATCTTTAGATAATTTAGACTGCATACTCAAATACATTTGATATCCTCCTTAGTTCCTATTCCG |
| 17978 *olsB*Ab confirm 5' | GTGGTGTTGTGAGCGCACATATTG |
| 17978 *olsB*Ab confirm 3' | GCCTTGAGTCGCCTTACGAATATG |
| 17978 *phoR*Ab Kan FRT 5' | CGTTTGCTAAACAAGATTTACGACTTTTATTATTTTTCCTGATTATTGCAGGTTTAGTCGGTTTAGGAATTGGGTATTTCTGGAGCTGTATTTTTATTGCCTTTGTGGTGTTTTTTACACTTCAGAGCGATTGTGTAGGCTGGAGCTGCTTCG |
| 17978 *phoR*Ab Kan FRT 3' | ATGTTATAGAGTCTTTCTTTTGGAAAAACTGCGGTAAAGGTTGATCCTTCATTTTCTTTAGATTGCACATCTAAGTAGGCGCCGTGTTGCATGAGTACATGTTTTACAATCGCCAAGCCTAAACCATATCCTCCTTAGTTCCTATTCCG |
| 17978 *phoR*Ab 5' confirm | GGACCAACAGAATACCGTCTGCTTG |
| 17978 *phoR*Ab 3' confirm | GATGGTGGAGATCATCGTGATGCAC |
|  |  |
| **Complementation Primers** |  |
| 17978 *olsB*Ab KpnI 5' | CGCGGTACCATGCTGGAAAAATTTAATCAATATCGC |
| 17978 *olsB*Ab SalI 3' | CGCGTCGACTTATCGCTGAGCCATTTTGTTC |
| 17978 *phoR*Ab SacI 5' | CGCGAGCTCATGTATGAACCCTACCCCGTCC |
| 17978 *phoR*Ab BamHI 3' | CGCGGATCCTTAAGTCATGTTATAGAGTCTTTCTTTTGG |
|  |  |
| **Transposon mutant primers** |  |
| AB5075 *olsB::tn101* 5' confirm | CGACACAAGAAGTTGACCGTTTAATCG |
| AB5075 *olsB::tn101* 3' confirm | CAATTGGTGATTCAACTGCAGACTGG |
| AB5075 *olsA*:*:tn101* 5' confirm | GCTCAAGAATATCTGCACCAGCTGAC |
| AB5075 *olsA::tn101* 3' confirm | GGATGAGCTGGCAACTAAAGCG |
| AB5075 *phoR::tn26* 5' confirm | GTCTGGATGCTGGTGCAGATGAC |
| AB5075 *phoR::tn26* 3' confirm | GGTGCCGACTGTACCGTTAATG |
